# Upper instrumented vertebrae selection criteria for degenerative lumbar scoliosis based on the hounsfield unit asymmetry of the first coronal reverse vertebrae: an observational study

**DOI:** 10.1186/s13018-023-04325-z

**Published:** 2023-11-01

**Authors:** Xiangyu Hou, Zhuoran Sun, Weishi Li, Hui Wang, Lin Zhuo, Lei Yuan, Yan Zeng, Linyao Ding, Ze Chen

**Affiliations:** 1 Department of Orthopaedics, Peking University Third Hospital, Haidian District, No 49. North Garden Road, Beijing, 100191 China; 2 Engineering Research Center of Bone and Joint Precision Medicine, Beijing, China; 3 Beijing Key Laboratory of Spinal Disease Research, Beijing, China; 4 Department of Orthopaedics, Third Hospital of Hebei Medical University, Shijiazhuang, China; 5 Research Center of Clinical Epidemiology, Peking University Third Hospital, Beijing, China

**Keywords:** First coronal reverse vertebra, Degenerative lumbar scoliosis, Upper instrumented vertebra, Hounsfield unit, Adjacent segment degeneration

## Abstract

**Background:**

Selection of the upper instrumented vertebra (UIV) is crucial for surgical treatment of degenerative lumbar scoliosis (DLS), given the relevance of UIV in postoperative proximal adjacent segment degeneration (pASD). Our previous research found that selection of UIV not lower than (≤) the first coronal reverse vertebra (FCRV), which marks the turning point of Hounsfield unit (HU) asymmetry, could significantly reduce pASD. However, the degree of HU asymmetry can vary among patients, suggesting a demand for more individualized UIV selection criteria, which we aimed to develop using quantitative HU measurement in the current study.

**Methods:**

We included 153 consecutive patients with DLS. Quantitative measurement of HU of both sides of the vertebrae of these patients was performed on three planes of CT reconstruction for average values and determination of FCRV. Pre- and postoperative X-ray plain films were examined for radiological measurements and determination of pASD. Further, 35 patients with lumbar disc herniation and without significant scoliosis were also included as the reference group, and their bilateral HU was measured.

**Results:**

In all 153 patients, those with UIV ≤ FCRV had a significantly lower rate of pASD (9.4% vs. 24.6%, *P* = 0.011). The difference between HU of the left and right sides of the FCRV (dF) could range from close to 0–59.4. The difference between HU of the left and right sides of the vertebrae in the reference group had an average value of 5.21. In 101 dF ≥ 5 DLS patients, those with UIV ≤ FCRV had a significantly lower rate of pASD (7.6% vs. 28.6%, *P* = 0.005), while this rate was insignificant in the other 52 dF < 5 patients (13.3% vs. 18.2%, *P* = 0.708). No other general, radiological, or operative parameter was found to have significant influence on the occurrence of pASD.

**Conclusions:**

Selection of UIV ≤ FCRV can significantly reduce the risk of pASD for patients with DLS with dF ≥ 5.

*Trial Registration* Not applicable, since this is an observational study.

## Background

Degenerative lumbar scoliosis (DLS) is increasing in incidence due to the rapidly ageing population [1]. Surgical treatment is required for severe DLS for which symptoms cannot be relieved with conservative treatment [1, 2]. Adjacent segment degeneration (ASD) is common after lumbar spinal fusion surgeries [3] and is about 50-fold more prevalent in DLS patients [5]. Because of its close relevance to proximal ASD (pASD) in DLS [8], the selection of the upper instrumented vertebra (UIV) is especially important during the surgical treatment of DLS.

Selection of UIV is conventionally performed through radiological identification of coronal stability in anteroposterior X-ray plain film. Previous studies have suggested the upper end vertebra (UEV), stable vertebra (SV) [9], horizontal vertebra (HV) [10], and neutral vertebra (NV) [11, 12] as possible bases of radiological selection of UIV, but there has not been any consensus. All of the above strategies depend on the position and orientation of the vertebrae, making them susceptible to posture during X-ray filming [12], thereby weakening their stability and reliability.

Our previous work demonstrated that selection of UIV that is not lower than the first coronal reverse vertebrae (FCRV) could significantly reduce pASD in DLS patients, and that the FCRV could be a more reliable selection criterion for UIV [13]. This has biomechanical bases. Bone mineral density (BMD) asymmetry in the DLS spine demonstrates higher HU within concavity and lower HU within convexity of the same vertebra [14] in computed tomography (CT) images, and FCRV is defined as the first, most caudal vertebra presenting opposite orientation of HU asymmetry to the vertebrae within the major curve when measuring the concave-convex HU of the vertebrae from caudal to cranial [14]. Theoretically, pASD is caused by the abnormally enlarged force loaded on the segment proximally adjacent to the UIV [15], meaning the biomechanical condition is crucial to UIV selection. HU can reflect the BMD and the biomechanical condition [16] of the vertebrae, which are therefore fundamental pathogenic characters. Considering the reversion of HU asymmetry and the close relationship between BMD and the regional biomechanical condition [16], it is rational to believe that the FCRV represents a biomechanical transitional region, possibly a force concentration area, and that the instrumentation should cross this area, which means that UIV should not be lower than FCRV. Moreover, the HU is independent of the posture of the patient when taking the CT image [17, 18], making the determination of FCRV more stable than those of the vertebrae determined on X-ray plain films.

However, the degree of HU asymmetry of the FCRV and its relationship with UIV selection has not yet been quantitatively described or investigated. Vertebral HU asymmetry can differ between the different regions of the DLS spine and different patients. The average ratio of HU between the concave and convex in different vertebrae can vary from 0.84 to 3.10 in groups of patients with different Cobb angles, and the difference between the average HU of the concave and convex can vary from 9.9 to 76.5 [14]. This indicates different biomechanical conditions, possibly suggesting a demand for more individualized UIV selection criteria.

This study aimed to investigate more individualized FCRV-based UIV selection criteria by quantitatively measuring the HU of both sides of the vertebrae of DLS patients.

## Methods

### Patients

This study was approved by the Institutional Review Board (Approval No.: IRB00006761-M2020291). The need for individual consent was waived. We enrolled DLS patients who underwent surgery in our hospital. Inclusion criteria: 1. over 45 years of age at the time of surgery, 2. a minimum follow-up of 2 years, with lumbar CT scan taken preoperatively and full spine standing anteroposterior and lateral X-ray plain films taken preoperatively, 3 months and 2 years postoperatively, 3. posterior instrumentation from the thoracolumbar region to the lower lumbar region or the sacrum, and 4. having gone through posterior column osteotomy including Ponte osteotomy or asymmetrical pedicle subtraction osteotomy. Exclusion criteria: 1. history of surgery in the thoracic or lumbar vertebrae, 2. spinal fractures, spinal infections or metabolic diseases that may potentially affect surgical outcome, and 3. anatomical identification too difficult for radiological measurement. Retrieval of the medical records from January 2010 to November 2018 identified 153 consecutive DLS patients who met the criteria for retrospective review.

We further included 35 lumbar disc herniation (LDH) patients without significant scoliosis or bony structural degeneration as the reference group for comparison of HU asymmetry, for measurement of the vertebral HUs.

### Clinical and radiological data

Patient demographics, including age and sex, were recorded. Perioperative parameters including UIV location, lower instrumented vertebra (LIV) location, and fusion level were reviewed and recorded.

Postoperative pASD was evaluated at a 2-year follow-up. Patients with either postoperative progression of proximal coronal degeneration (PCD) or sagittal proximal junctional kyphosis (PJK) were diagnosed with pASD. PCD was defined by the presence of any of the following criteria by comparison of the 2-year follow-up and the postoperative anteroposterior radiographs: progression of disc wedging of the intervertebral disc between UIV and the vertebra above UIV (UIV-1) over 5°, coronal proximal junctional angle (PJA, the cobb angle between the lower endplate of the UIV and the upper endplate of the UIV-2 [19]) progression of over 10°, progression of the obliquity of UIV-1 upper endplate over 10°, lateral translation between UIV-1 and UIV over 3 mm, change of coronal balance distance (CBD) over 30 mm, or osteophyte growth of the UIV or UIV-1 over 3 mm. PJK was defined by the presence of both sagittal PJA ≥ 10° and sagittal PJA > 10° more than the postoperative measurement.

Preoperative and postoperative radiographic parameters were also measured. The Cobb angle of the major lumbar curve, disc wedging above UIV, upper endplate obliquity, coronal PJA, lateral translation between UIV-1 and UIV, CBD, and osteophyte growth were measured on the anteroposterior radiographs. Pelvic incidence (PI), pelvic tilt (PT), thoracic kyphosis (TK), thoracolumbar kyphosis (TLK), lumbar lordosis (LL), sagittal vertical axis (SVA), and sagittal PJA were measured on lateral X-ray plain films.

**Fig. 1:**
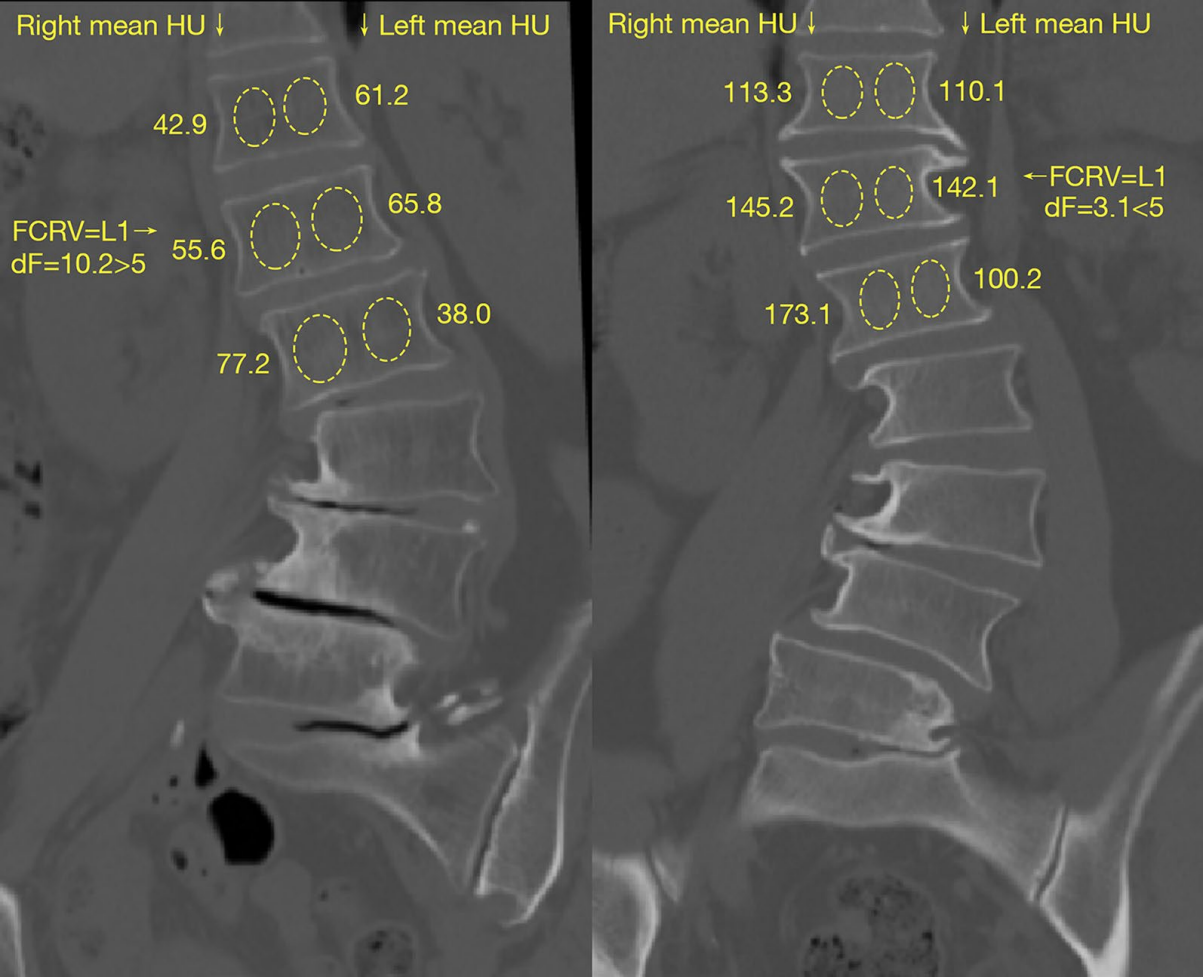
The coronal reconstruction images of the preoperative lumbar CT scans of a dF > 5 patient (**a**) and a dF < 5 patient (**b**). The circles show the ROIs measuring HU on one of the 3 slices, while the numbers beside them show the mean HU from the 3 slices. The FCRV marks the turning point of HU asymmetry

The HU was measured following the protocol of Wang et al. [14], and the differences between the HU of the Left and Right Sides of the FCRV (dF) were calculated with the measured HU. Regions of interest (ROI) were selected on the coronal reconstruction of the preoperative CT at 3 locations: immediately posterior to the anterior margin of the vertebrae, in the middle of the vertebral body, and anterior to the posterior margin of the vertebrae (not too posterior, to avoid blood vessels). The ROIs were carefully chosen to avoid cortical bone or bony islands. HU measurements within the concave and convex sides of the vertebrae were obtained both from the UIV-1 (the vertebra 1 segment cranial to the UIV) to UIV + 1 (the vertebra 1 segment caudal to the UIV) and from FCRV-1 to FCRV + 1. The HU from the 3 coronal slices was averaged for a mean HU for both sides of each vertebra. FCRV was defined as the first (most caudal) vertebra presenting opposite orientation of HU asymmetry from the apical vertebra (Fig. 1).

For LDH patients without significant scoliosis, HU in the vertebrae from T11 to L2 were measured in the same way on both sides, as the FCRV appear most frequently in this region [13]. The HU of 208 sides in 104 vertebrae was measured in the same way as for DLS patients. These patients were set as the reference group, and the difference between the HU of two sides of their vertebrae was calculated and evaluated for comparison with DLS patients.

### Statistical analysis

Data were analysed using Statistical Product and Service Solutions software (version 26.0; SPSS, Chicago, IL). Continuous variables were recorded as mean ± standard deviation, and categorical variables were expressed as frequency or percentages. An independent *t*-test or one-way analysis of variance (ANOVA) was used to analyse the difference between continuous variables. χ^2^ analysis and Fisher’s exact test were used to examine differences among categorical variables. Logistic regression was used to rule out other confounding factors. Statistical significance was set at *P* < 0.05.

## Results

### Patient data

Among 153 patients, 120 are female and 33 are male, with a mean age of 63.57 ± 6.58 years. At a 2-year follow-up, 23/153 (15.0%) presented radiological pASD, while 130 (85%) presented no radiological pASD. Among the 23 patients with pASD, 11 had PJK, 17 had PCD, and 5 had both. Further, 96/153 (62.7%) had UIV equal to or higher than the FCRV (UIV ≤ FCRV), while 57 (37.3%) had UIV lower than the FCRV (UIV > FCRV).

### Comparison of general, radiological, and operative data between patients with and without pASD

General, radiological, and operative parameters of patients with and without pASD are shown in Table 1. There was no significant difference in sex, age, or preoperative radiological parameters including dF, Cobb angle, CBD, PI, PT, TK, LL, and SVA, between patients with and without pASD. Regarding operative parameters, UIV and fusion level showed no significant difference, nor was there any difference whether the LIV was on L5 or the sacrum. No statistically significant difference was found in postoperative radiological parameters including Cobb angle, PI, PT, TK, TLK, LL, and SVA. The average postoperative CBD was significantly larger in patients with than without pASD (*P* = 0.022).

**Table 1:** Comparison of general, radiological, and operative parameters between patients with and without pASD

	no pASD	pASD	P value
Number of patients	130	23	
Sex (female/male)	103/27	17/6	0.568
Age (years)	63.85 ± 6.55	61.96 ± 6.67	0.382
Preoperative dF (HU)	12.31 ± 12.15	8.17 ± 6.73	0.115
Preoperative Cobb angle (°)	29.51 ± 10.10	29.45 ± 13.03	0.979
Preoperative CBD (mm)	18.06 ± 16.31	20.88 ± 15.48	0.442
Preoperative PI (°)	51.45 ± 11.56	47.06 ± 9.60	0.360
Preoperative PT (°)	25.88 ± 9.73	24.05 ± 9.22	0.973
Preoperative TK (°)	20.79 ± 15.17	20.62 ± 11.71	0.960
Preoperative LL (°)	24.96 ± 20.98	22.17 ± 16.64	0.546
Preoperative SVA (mm)	50.78 ± 48.48	46.78 ± 51.74	0.719
UIV	11.92 ± 1.37	12.35 ± 1.80	0.195
Lower instrumented vertebra (L5/sacrum)	60/70	10/13	0.812
Fusion level	5.63 ± 1.54	5.22 ± 1.91	0.254
Postoperative Cobb Angle (°)	11.42 ± 6.77	13.10 ± 6.86	0.286
Postoperative CBD (mm)	18.89 ± 15.48	11.03 ± 9.14	0.022*
Postoperative PI (°)	48.36 ± 10.27	46.20 ± 10.43	0.373
Postoperative PT (°)	19.81 ± 8.70	17.97 ± 8.25	0.435
Postoperative TK (°)	21.87 ± 10.93	27.42 ± 11.56	0.777
Postoperative TLK (°)	8.02 ± 9.89	14.06 ± 10.53	0.893
Postoperative LL (°)	21.07 ± 34.01	35.14 ± 25.10	0.066
Postoperative SVA (mm)	28.26 ± 43.84	15.35 ± 36.41	0.224

^*^P < 0.05

### Relationship between UIV selection and pASD

To evaluate the value of FCRV in UIV selection, we divided patients into the UIV ≤ FCRV group and the UIV > FCRV group. In total, 9/96 (9.4%) and 14/57 (24.6%) patients in the UIV ≤ FCRV and UIV > FCRV groups developed pASD, respectively. The UIV ≤ FCRV group had a significantly lower rate of development of pASD (*P* = 0.011) (Table 2).

**Table 2:** The relationship between UIV selection and pASD in all DLS patients

	UIV > FCRV	UIV ≤ FCRV	Total
pASD	14 (24.6%)	9 (9.4%)	23 (15.0%)
no pASD	43 (75.4%)	87 (90.6%)	130 (85.0%)
Total	57	96	153

*P* = 0.011

Considering that the average postoperative CBD was significantly different between patients with and without pASD, logistic regression was used to analyse the influence of both the relationship between UIV and FCRV and the postoperative CBD on pASD. The result showed that the relationship between UIV and FCRV significantly influenced pASD occurrence (*P* = 0.020), while postoperative CBD had no significant influence (*P* = 0.061).

### HU difference on the left and right sides of the FCRV (dF) among DLS patients

In the 153 DLS patients, dF varied from almost 0 to 59.4. The average value was 11.68, the median value was 8.67, and the standard deviation was 11.57. About 1/3 (52) of the patients had dF < 5.

### HU difference on the left and right sides of the vertebrae in LDH patients without significant scoliosis (reference group)

Overall, 208 sides of 104 vertebrae from 35 LDH patients without significant scoliosis underwent HU measurement of the T10 to L2 vertebrae using the same method as DLS patients. The difference between the HU of the left and right sides of the vertebrae ranged from 0 to 18.4. The average value was 5.21, the median value was 4.20, and the standard deviation was 4.32.

### Differences between patients with different dF

The relationship between UIV and FCRV showed a different influence on the development of pASD in patients with different degrees of HU asymmetry in the FCRV. The degree of HU asymmetry was measured by dF (Fig. 1), and patients were divided into dF < 5 (n = 52) and dF ≥ 5 groups (n = 101). General, radiological, and operative parameters of the two groups are shown and compared in Table 3.

**Table 3:** Comparison of general, radiological, and operative parameters between the dF < 5 group and the dF ≥ 5 group

	dF < 5	dF ≥ 5	P value
preoperative dF (HU)	2.45 ± 1.28	16.24 ± 11.68	< 0.001*
Number of Patients	52	101	
Sex (female/male)	38/14	82/19	0.248
Age (years)	63.56 ± 6.46	63.57 ± 6.67	0.896
pASD occurrence (pASD / no pASD)	8/44	15/86	0.930
preoperative Cobb angle (°)	28.31 ± 10.01	30.11 ± 10.80	0.318
preoperative CBD (mm)	19.25 ± 21.33	18.08 ± 12.84	0.674
preoperative PI (°)	51.00 ± 12.57	50.68 ± 10.76	0.142
preoperative PT (°)	27.56 ± 9.12	24.60 ± 9.80	0.444
preoperative TK (°)	20.91 ± 14.09	20.69 ± 15.03	0.801
preoperative LL (°)	24.50 ± 15.39	24.56 ± 22.58	0.984
preoperative SVA (mm)	58.59 ± 51.56	45.85 ± 47.03	0.127
UIV	11.90 ± 1.59	12.03 ± 1.37	0.612
FCRV	11.94 ± 1.75	12.00 ± 1.13	0.806
UIV ≤ FCRV / UIV > FCRV	30/22	66/35	0.357
lower instrumented vertebra (L5/sacrum)	23/29	47/54	0.786
fusion level	5.65 ± 1.67	5.52 ± 1.57	0.637
postoperative Cobb angle (°)	10.99 ± 7.19	12.01 ± 6.58	0.383
postoperative CBD (mm)	15.85 ± 15.24	18.71 ± 14.80	0.268
postoperative PI (°)	47.05 ± 11.28	48.56 ± 9.76	0.401
postoperative PT (°)	20.67 ± 10.11	18.97 ± 7.82	0.149
postoperative TK (°)	24.60 ± 11.06	21.89 ± 11.18	0.259
postoperative TLK (°)	12.02 ± 10.58	6.95 ± 9.44	0.616
postoperative LL (°)	23.15 ± 31.62	23.09 ± 34.11	0.991
postoperative SVA (mm)	29.29 ± 52.11	25.14 ± 37.65	0.583

^*^P < 0.05

The average preoperative dF in the dF < 5 and dF ≥ 5 groups were 2.45 and 16.24, respectively. There was no significant difference in sex, age, occurrence of pASD, preoperative radiological parameters, including Cobb angle, CBD, PI, PT, TK, LL, and SVA, operative radiological parameters, including UIV, LIV, and fusion level, and postoperative radiological parameters, including Cobb angle, CBD, PI, PT, TK, TLK, LL, and SVA, between the two groups (Table 3).

**Fig. 2:**
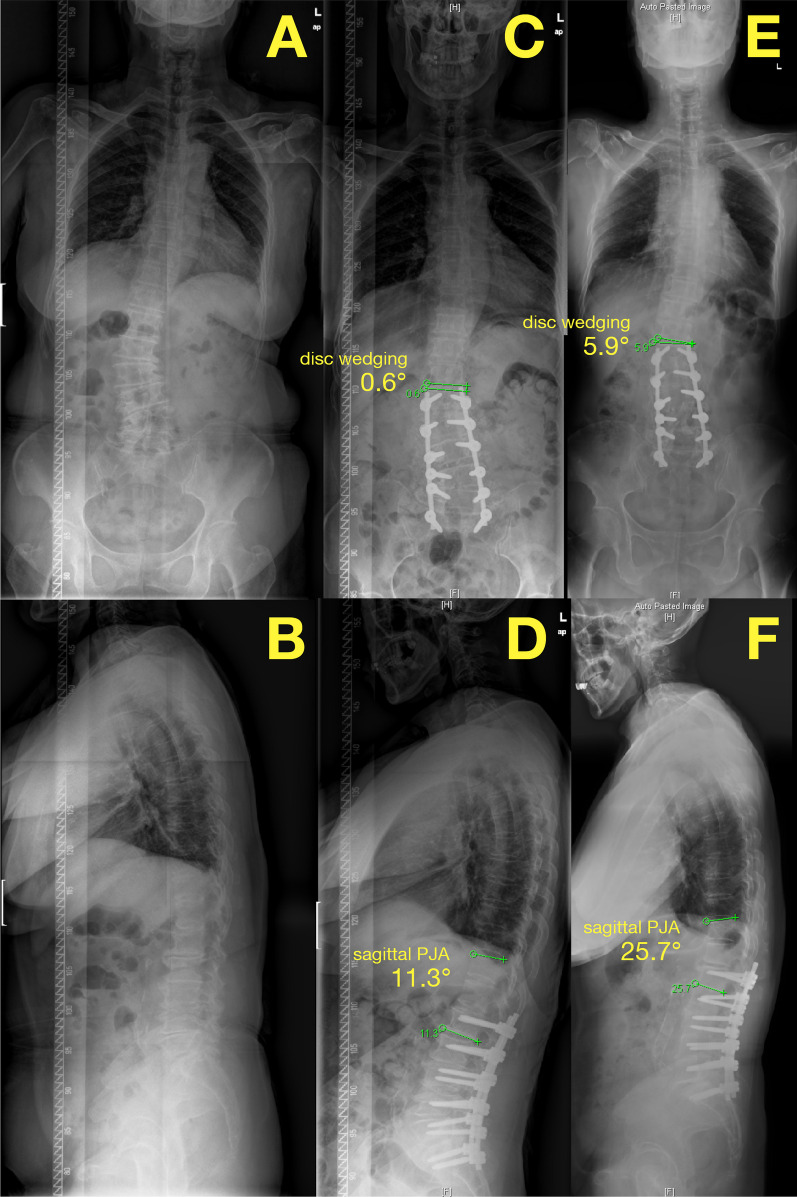
The preoperative (**a**, **b**), postoperative (**c**, **d**), and 2-year follow-up (**e**, **f**) X-ray plain films of a dF > 5 patient with UIV > FCRV (FCRV = T11, UIV = L1). The patient had pASD of both PCD (**c**, **e**, progression of disc wedging of 5.3°) and PJK (**d**, **f**, progression of sagittal PJA of 14.4°)

**Fig. 3:**
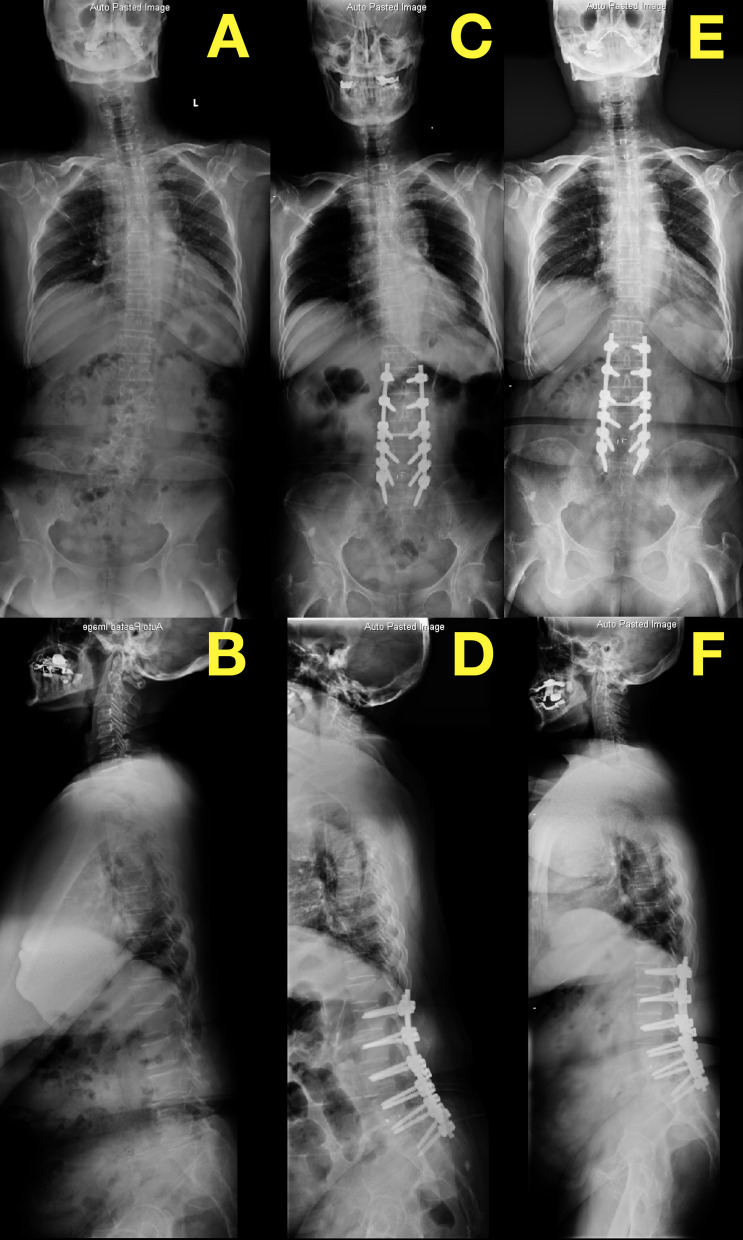
The preoperative (**a**, **b**), postoperative (**c**, **d**), and 2-year follow-up (**e**, **f**) X-ray plain films of a dF > 5 patient with UIV ≤ FCRV (FCRV = UIV = L1). The patient had no pASD

In the dF < 5 group, no significant difference in pASD (*P* = 0.708) was noticed between UIV ≤ FCRV and UIV > FCRV patients (Table 4). In the dF ≥ 5 group, however, the difference was obviously significant (*P* = 0.005), with a total pASD of 10/35 (28.6%) in the UIV > FCRV group (Fig. 2), compared with 5/66 (7.6%) in the UIV ≤ FCRV group (Fig. 3) (Table 5).

**Table 4:** The relationship between UIV selection and pASD in dF < 5 patients

	UIV > FCRV	UIV ≤ FCRV	Total
pASD	4 (18.2%)	4 (13.3%)	8 (15.4%)
no pASD	18 (81.8%)	26 (86.7%)	44 (84.6%)
Total	22	30	52

*P* = 0.708

**Table 5:** The relationship between UIV selection and pASD in dF ≥ 5 patients

	UIV > FCRV	UIV ≤ FCRV	Total
pASD	10 (28.6%)	5 (7.6%)	15 (14.9%)
no pASD	25 (71.4%)	61 (92.4%)	86 (85.1%)
Total	35	66	101

*P* = 0.005

## Discussion

The FCRV in DLS is the most caudal vertebra cranial to the apical vertebra, representing HU asymmetry opposite from the main curve (Fig. 1). Patients with UIV ≤ FCRV had a significantly lower rate of pASD development, while pASD was not significantly influenced by general, radiological, or surgical parameters other than the relationship between UIV and FCRV, as shown in our prior research [13] and the present study (Tables 1 and 2). These can prove the value of FCRV in clinical decisions for DLS patients. However, the determination of FCRV is qualitative, regardless of the exact dF. The dF varied from near 0 to 59.4 in the present study, indicating varied biomechanical conditions, and potentially demanding different UIV selection criteria in patients with different dF.

HU is closely related to the biomechanical conditions of the vertebrae. Measuring HU from CT scans has been proven as a robust technique for assessing bone quality inside the vertebral bodies, with reliability and accuracy independent of posture [17, 18]. According to Wolff’s law [16], the regions bearing larger force have higher BMD for adaptation. The HUs of polyurethane bricks have been proven to be linearly correlated with the plastic modulus [20]. The BMD calculated using HU also has a mathematical relationship close to linear with the loading force [21].

Asymmetrical hydrostatic pressure has been observed in the vertebral discs of scoliotic spines by intraoperative pressure measurement [22]. Asymmetrical loading force can accelerate vertebral degeneration by affecting both the bony structures and the soft tissues. The degree of cellular necrosis is positively correlated with the degree of pressure asymmetry [22, 23]. Coronally asymmetrical pressure on the vertebral disc can trigger the nucleus pulposus to lose its mechanical homogeneity, thereby posing abnormally enlarged regional pressure to the annulus fibrosus [22]. This accelerates cell death and disc and vertebra degeneration [23]. Asymmetrical load may also cause the bony structures to grow asymmetrically in the longitudinal direction, thereby wedging the vertebral bodies and intervertebral discs [24].

These results suggest that the degree of HU asymmetry, represented by dF in the FCRV, is related to the degree of pressure asymmetry and, thus, the degeneration of the vertebrae. As such, quantitative measurement of HU may have significant potential in assessing the biomechanical condition of the vertebrae and assisting UIV selection, and patients with different dF may require different UIV selection criteria. In the present study, by grouping the patients according to their degree of HU asymmetry in the FCRV, we further discovered that the simple “UIV ≤ FCRV” criterion showed different clinical efficacy in patients with different dF. The threshold value for sufficient asymmetry was determined through comparison with LDH patients without significant scoliosis, with an average difference of HU between the left and right sides of the T10-L2 vertebrae (where the FCRV most frequently locates) of 5.21, and a median value of 4.20. Therefore, 5 was set as the threshold, as an HU asymmetry less than that of an average patient with no significant scoliosis cannot be rationally considered sufficient. In patients with a dF < 5, the original UIV ≤ FCRV criterion showed little clinical value, with no statistically significant difference in the occurrence of pASD between the UIV ≤ FCRV and UIV > FCRV groups (Table 4). In dF ≥ 5 patients, however, the UIV ≤ FCRV criterion shows significant value in UIV selection, where the UIV ≤ FCRV group has significantly less pASD (Table 5). This shows that the “UIV ≤ FCRV” criterion cannot be simply applied to all DLS patients and that patients with dF ≥ 5 represent an area of application better than all DLS patients. The irrelevance of dF for the preoperative general and radiological features like sex, age, Cobb, PI, SS, and SVA further indicates its uniqueness (Table 3).

The study has some limitations. First, manual ROI selection torsion and obliquity of the vertebrae, and severe degeneration involving osteophytes, bony islands, and severe osteoporosis, may have introduced errors in HU measurement in DLS patients. To reduce errors, we used the average of measurements at 3 locations and avoided bony islands while choosing the ROI. We believe that more accurate HU measuring methods may be developed in the future. Second, 5 has not been strictly proven to be the optimal threshold for determining sufficient HU asymmetry. During this research, we found that this threshold could not be determined simply with statistical methods, so we measured the HU of LDH patients with no significant scoliosis as a reference. Though it is not a perfect method and may require larger samples for further correction, it was adopted considering the convenience in clinical practice and the sufficient clinical efficacy in the present study. Third, the present study only reveals that the “UIV ≤ FCRV” criterion is optimal for dF ≥ 5 patients. However, the UIV selection criteria for dF < 5 patients, constituting ~ 1/3 of DLS patients, remain unclear, on which future studies may focus.

## Conclusions

In conclusion, the selection of UIV not lower than FCRV can significantly reduce the risk of proximal ASD in DLS for patients with HU differences between the left and right sides of the FCRV of not less than 5.

## Data Availability

The data generated and analysed during the current study are not publicly available because they will also be used in the future researches, but are available from the corresponding author on reasonable request.

## Abbreviations

DLS: Degenerative lumbar scoliosis
UIV: Upper instrumented vertebra
FCRV: First coronal reverse vertebra
HU: Hounsfield unit
ASD: Adjacent segment degeneration
pASD: Proximal adjacent segment degeneration
UEV: Upper end vertebra
SV: Stable vertebra
HV: Horizontal vertebra
NV: Neutral vertebra
BMD: Bone mineral density
CT: Computed tomography
LDH: Lumbar disc herniation
PCD: Proximal coronal degeneration
PJK: Proximal junctional kyphosis
PJA: Proximal junctional angle
CBD: Coronal balance distance
PI: Pelvic incidence
PT: Pelvic tilt
TK: Thoracic kyphosis
TLK: Thoracolumbar kyphosis
LL: Lumbar lordosis
SVA: Sagittal vertical axis
dF: Difference between the HU of the Left and Right Sides of the FCRV
ROI: Region of interest
ANOVA: Analysis of variance

## References

[1] Aebi M (2005). The adult scoliosis. Eur Spine J.

[2] Cho KJ, Kim YT, Shin SH, Suk SI (2014). Surgical treatment of adult degenerative scoliosis. Asian Spine J.

[3] Ghiselli G, Wang JC, Bhatia NN, Hsu WK, Dawson EG (2004). Adjacent segment degeneration in the lumbar spine. J Bone Joint Surg Am.

[4] Wang H, Ma L, Yang D, Wang T, Yang S, Wang Y (2016). Incidence and risk factors for the progression of proximal junctional kyphosis in degenerative lumbar scoliosis following long instrumented posterior spinal fusion. Medicine.

[5] Alentado VJ, Lubelski D, Healy AT, Orr RD, Steinmetz MP, Benzel EC (2016). Predisposing characteristics of adjacent segment disease after lumbar fusion. Spine.

[6] Lee N, Yi S, Shin DA, Kim KN, do Yoon H, Ha Y (2016). Progression of coronal cobb angle after short-segment lumbar interbody fusion in patients with degenerative lumbar stenosis. World Neurosurg..

[7] Burch MB, Wiegers NW, Patil S, Nourbakhsh A (2020). Incidence and risk factors of reoperation in patients with adjacent segment disease: a meta-analysis. J Craniovertebr Junction Spine.

[8] Phan K, Xu J, Maharaj MM, Li J, Kim JS, Di Capua J (2017). Outcomes of short fusion versus long fusion for adult degenerative scoliosis: a systematic review and meta-analysis. Orthop Surg.

[9] Bridwell KH (2004). Selection of instrumentation and fusion levels for scoliosis: where to start and where to stop. Invited submission from the Joint Section Meeting on Disorders of the Spine and Peripheral Nerves. J Neurosurg.

[10] Simmons ED (2001). Surgical treatment of patients with lumbar spinal stenosis with associated scoliosis. Clin Orthop Relat Res.

[11] Ha KY, Kim YH, Ahn JH (2014). Is it real adjacent segment pathology by stress concentration after limited fusion in degenerative lumbar scoliosis?. Spine.

[12] Cho KJ, Suk SI, Park SR, Kim JH, Jung JH (2013). Selection of proximal fusion level for adult degenerative lumbar scoliosis. Eur Spine J.

[13] Wang H, Sun Z, Wang L, Zou D, Li W (2023). Proximal fusion level above first coronal reverse vertebrae: an essential factor decreasing the risk of adjacent segment degeneration in degenerative lumbar scoliosis. Global Spine J.

[14] Wang H, Zou D, Sun Z, Wang L, Ding W, Li W. Hounsfield unit for assessing vertebral bone quality and asymmetrical vertebral degeneration in degenerative lumbar scoliosis. Spine. 2020. 10.1097/BRS.0000000000003639 32756284

[15] Hilibrand AS, Robbins M (2004). Adjacent segment degeneration and adjacent segment disease: the consequences of spinal fusion?. Spine J.

[16] Johnson KA (2014). Wolff's law continues to inspire orthopaedic research. Vet Comp Orthop Traumatol..

[17] Choi MK, Kim SM, Lim JK (2016). Diagnostic efficacy of Hounsfield units in spine CT for the assessment of real bone mineral density of degenerative spine: correlation study between T-scores determined by DEXA scan and Hounsfield units from CT. Acta Neurochir.

[18] Lee S, Chung CK, Oh SH, Park SB (2013). Correlation between bone mineral density measured by dual-energy X-ray absorptiometry and hounsfield units measured by diagnostic ct in lumbar spine. J Korean Neurosurg Soc.

[19] Glattes RC, Bridwell KH, Lenke LG, Kim YJ, Rinella A, Edwards C (2005). Proximal junctional kyphosis in adult spinal deformity following long instrumented posterior spinal fusion: incidence, outcomes, and risk factor analysis. Spine.

[20] Schreiber JJ, Anderson PA, Rosas HG, Buchholz AL, Au AG (2011). Hounsfield units for assessing bone mineral density and strength: a tool for osteoporosis management. J Bone Joint Surg Am.

[21] Gutekunst DJ, Patel TK, Smith KE, Commean PK, Silva MJ, Sinacore DR (2013). Predicting ex vivo failure loads in human metatarsals using bone strength indices derived from volumetric quantitative computed tomography. J Biomech.

[22] Meir AR, Fairbank JC, Jones DA, McNally DS, Urban JP (2007). High pressures and asymmetrical stresses in the scoliotic disc in the absence of muscle loading. Scoliosis.

[23] Lotz JC, Chin JR (2000). Intervertebral disc cell death is dependent on the magnitude and duration of spinal loading. Spine.

[24] Mente PL, Stokes IA, Spence H, Aronsson DD (1997). Progression of vertebral wedging in an asymmetrically loaded rat tail model. Spine.

